# MicroRNA expression profile in serum reveals novel diagnostic biomarkers for endometrial cancer

**DOI:** 10.1042/BSR20210111

**Published:** 2021-06-16

**Authors:** Xingchen Fan, Xuan Zou, Cheng Liu, Wenfang Cheng, Shiyu Zhang, Xiangnan Geng, Wei Zhu

**Affiliations:** 1 Department of Oncology, First Affiliated Hospital of Nanjing Medical University, 300 Guangzhou Road, Nanjing 210029, P.R. China; 2 Department of Medical Oncology, Fudan University Shanghai Cancer Center, Shanghai 200032, P.R. China; 3 Department of Oncology, Shanghai Medical College, Fudan University, Shanghai 200032, P.R. China; 4 Department of Gastroenterology, First Affiliated Hospital of Nanjing Medical University, 300 Guangzhou Road, Nanjing 210029, P.R. China; 5 Department of Clinical Engineer, First Affiliated Hospital of Nanjing Medical University, 300 Guangzhou Road, Nanjing 210029, P.R. China

**Keywords:** diagnostic biomarker, endometrial cancer, qRT-PCR, serum microRNA

## Abstract

**Purpose:** Circulating microRNAs (miRNAs) prove to be promising diagnostic biomarkers for various cancers, including endometrial cancer (EC). The present study aims to identify serum microRNAs that can serve as potential biomarkers for EC diagnosis.

**Patients and methods:** A total of 92 EC and 102 normal control (NC) serum samples were analyzed using quantitative real-time polymerase chain reaction (qRT-PCR) in this four-phase experiment. The logistic regression method was used to construct a diagnostic model based on the differentially expressed miRNAs in serum. The receiver operating characteristic (ROC) curve analysis was performed to evaluate the diagnostic value. To further validate the diagnostic capacity of the identified signature, the 6-miRNA marker was compared with previously reported biomarkers and verified in three public datasets. In addition, the expression characteristics of the identified miRNAs were further explored in tissue and serum exosomes samples.

**Results:** Six miRNAs (miR-143-3p, miR-195-5p, miR-20b-5p, miR-204-5p, miR-423-3p, and miR-484) were significantly overexpressed in the serum of EC compared with NCs. Areas under the ROC of the 6-miRNA signatures were 0.748, 0.833, and 0.967 for the training, testing, and the external validation phases, respectively. The identified signature has a very stable diagnostic performance in the large cohorts of three public datasets. Compared with previously identified miRNA biomarkers, the 6-miRNA signature in the present study has superior performance in diagnosing EC. Moreover, the expression of miR-143-3p and miR-195-5p in tissues and the expression of miR-20b-5p in serum exosomes were consistent with those in serum.

**Conclusions:** We established a 6-miRNA signature in serum and they could function as potential non-invasive biomarker for EC diagnosis.

## Introduction

Endometrial cancer (EC) is one of the most frequent female malignancies and the fourth leading cause of cancer-related deaths among women worldwide [1,2]. Nearly 74000 women were expected to die of EC each year [3]. In China, approximately 50000 new cases of EC are added each year, and the number of deaths is 18000. With environmental pollution and increasing life stress in populations, the incidence of EC is growing rapidly [4,5]. Most guidelines recommend either transvaginal ultrasonography or endometrial biopsy as the initial test, but none of them are completely satisfactory. Diagnostic curettage is a widely used diagnostic method in EC patients. However, diagnostic curettage has several inherent defects, such as invasive nature. Beyond that, diagnostic curettage needs to be operated by experienced gynecologists and has a risk of missed diagnosis. Circulating biomarkers allow the composite analysis of tumors without the need for biopsy, surgery, or other invasive procedures. Despite the increasing efforts to identify reliable biomarkers, no specific serum markers have shown satisfactory EC diagnosis or monitoring performance.

MicroRNA (miRNA) is a small non-coding RNA that binds to target messenger ribonucleic acid (mRNA) to inhibit post-transcriptional gene expression and plays an essential role in regulating gene expression, cell cycle, biological development timing etc. [6]. Many studies have revealed its role in the biological processes of various cancers, helping to detect the existence of cancer diseases as early as possible, and screen out undiagnosed suspicious cases and healthy people [7–9]. Several studies have shown that specific miRNAs can be used as high-precision biomarkers for EC detection [10–12]. Meanwhile, circulating miRNAs show great potential of being cancer biomarkers for the stable existence in peripheral serum or plasma. Since blood samples are easy to obtain and the testing procedure is more convenient and cheaper, circulating biomarker can be used alone or combined with other traditional screening methods for preliminary screening before further invasive pathological and imaging examinations. It can help to identify the existence of cancer disease as early as possible, which can be employed for the screening of both undiagnosed suspected cases and healthy population. However, findings lack consistency due to demographic variables and methodological differences across studies. Systematic studies with larger research cohorts and more precise research methods are needed to discover more reliable biomarkers. In the current experiment, a four-phase study based on quantitative real-time polymerase chain reaction (qRT-PCR) was determined to identify serum miRNAs for the detection of EC. In addition, this experiment also analyzed the expression of miRNA in EC tissues and serum exosomes to facilitate the understanding of the possible forms and biological functions of the identified miRNAs.

## Materials and methods

### Study design and study population

A total of 92 patients diagnosed with EC and 102 normal female controls (NCs) were enrolled from the First Affiliated Hospital of Nanjing Medical University and Women’s Hospital of Nanjing Medical University between 2016 and 2017 in the present study. The present study has been approved by the institutional ethics committee and the patients written informed consent has been obtained (ID: 2016-SRFA-148). None of the patients received any treatment such as surgery, radiotherapy, or drug treatment before sampling. The demographics and clinical features of the patients were listed in Table 1. The criteria for sample inclusion have been summarized in Supplementary Table S1. The controls were matched to the patients by age and ethnicity.

**Table 1:** Clinical characteristics of 92 EC patients and 102 NCs

Variables	Training stage (*n*=45)		Testing stage (*n*=89)		External validation stage (*n*=60)	
	Cases (%)	Controls (%)	Cases (%)	Controls (%)	Cases (%)	Controls (%)
**Number**	21	24	41	48	30	30
**Age (years)**						
≤50	8 (38.1)	13 (64.5)	11 (26.8)	31 (64.6)	8 (26.7)	17 (56.7)
>50	13 (61.9)	11 (35.5)	30 (73.1)	17 (35.4)	22 (73.3)	13 (43.3)
**FIGO stage**						
I	19 (90.5)		39 (95.1)		27 (90.0)	
II, III, IV	2 (9.5)		2 (4.9)		3 (30.0)	
**Histological grade**						
G1	11 (52.4)		17 (41.5)		8 (26.7)	
G2	9 (42.8)		21 (51.2)		14 (46.6)	
G3	1 (4.8)		3 (7.3)		8 (26.7)	

Whole venous blood samples (5 ml) were collected using SST Advance Tubes (Becton, Dickinson and Company, New Jersey, U.S.A.). Serum samples were obtained following the centrifuging process of 3000 relative centrifugal force (RCF) for 10 min and then 12000 RCF for 2 min within 6 h. The obtained serum samples were stored in RNase-free tubes at −80°C until analysis. Tissue specimens were kept in liquid nitrogen.

**Figure 1:**
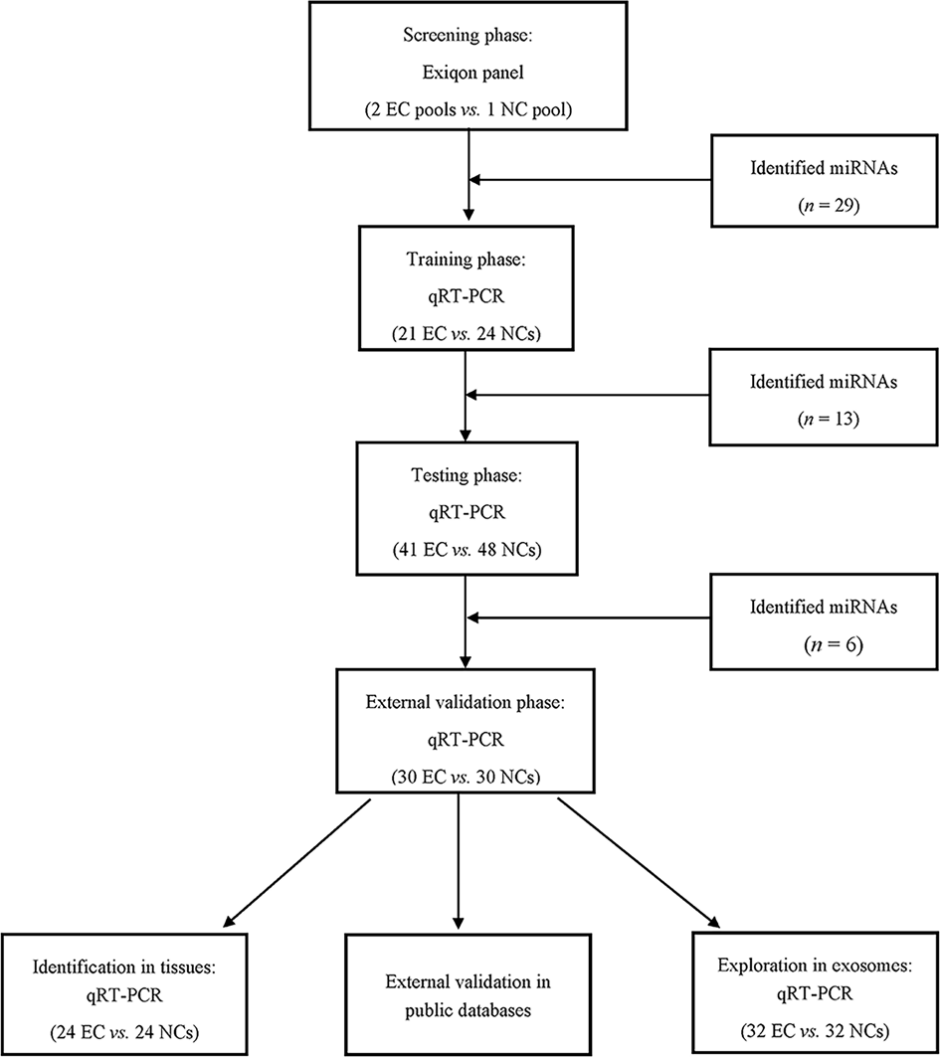
The flow chart of the experiment design

A multiphase study to identify a serum miRNA profile for screening EC is shown in Figure 1. During the initial screening phase, 2 pooled serum samples from 20 EC patients and 1 pooled sample from 10 NCs were selected randomly, and Exiqon miRCURY-Ready-to-Use PCR-Human-panel-I+II-V1.M (Exiqon miRNA qPCR panel, Vedbaek, Denmark) was applied to identify candidate miRNAs whose expression was altered in EC samples. In the training phase, 21 EC patients and 24 NCs were then performed to confirm the dysregulated miRNAs assessed by the screening phase. Then, the serum samples from 41 EC patients and 48 NCs were further analyzed during the testing phase to determine the differentially expressed miRNAs by qRT-PCR. In the external verification phase, 60 serum samples (30 EC vs. 30 NCs) were analyzed for verification.

### Isolation of exosomes

We used ExoQuick™ Exosome Precipitation Solution (System Biosciences, California, U.S.A.) to isolate exosomes from serum samples according to the manufacturer’s protocol. Exosomes pellet precipitated from 200 μl serum and 50 μl ExoQuick exosome precipitation solution was lysed in 200 μl RNase-free water until use.

### RNA extraction

According to the manufacturer’s protocol, RNA was isolated from 200 μl serum using mirVana Paris Kit (Ambion, Austin, TX, U.S.A.). Each sample was mixed with denaturing solution (Ambion, Austin, TX, U.S.A.) and then spiked with 5 μl synthetic *Caenorhabditis elegans* miRNA cel-miR-39 (5 nM/L, RiboBio, Guangzhou, China) to normalize sample-to-sample variation. Total RNA was extracted from Formalin-Fixed Paraffin-Embedded (FFPE) sample using the High Pure FFPE RNA Micro Kit (Ambion, Austin, TX, U.S.A.). Total RNA was eluted with 100 μl of RNase-free water and stored at −80°C for further experiments. The concentration and purity of RNA were analyzed by the Nanodrop 2000 spectrophotometer (NanoDrop Technologies, Wilmington, DE, U.S.A.) (A_260_/A_280_ = 1.8−2, A_260_/A_230_ > 1.7).

### qRT-PCR

The present study used Bulge-Loop™ miRNA qRT-PCR primer set (RiboBio, Guangzhou, China), which includes the specific primers of reverse transcription (RT) and polymerase chain reaction (PCR) to amplify miRNA. RT reactions were carried out at 70°C for 10 min and then 42°C for 60 min. The quantification of PCR product was evaluated based on the level of fluorescence emitted by SYBR Green (SYBR® Premix ExTaq™ II, TaKaRa). MiRNAs were amplified and detected in a Light Cycler 480 (Roche 480, Germany) real-time thermal cycler at a procedure of 95°C for 20 s, followed by 40 cycles of 95°C for 10 s, 60°C for 20 s, and then 70°C for 10 s. The expression levels of miRNAs in serum and exosomes were calculated using the 2^−ΔΔ*C*_t_^ method [13] (cel-miR-39 as exogenous reference miRNA; RNU6B[U6] for tissue sample normalization; Δ*C*_t_ = *C*t_miRNA_ − *C*t_normalizer_; *C*_t_, the threshold cycle).

### Statistical analysis

A non-parametric test (Mann–Whitney test) was used to compare miRNAs’ expression in EC patients and control subjects. A chi-squared test was used to analyze the distribution of the clinical features of CC patients. Logistic regression analysis was used to establish the miRNA panel. Then, the receiver operating characteristic (ROC) curves and the area under the ROC curve (AUC) were used to evaluate the sensitivity and specificity of identified miRNAs or the miRNA panels for EC detection. We have performed the decision curve analysis (DCA). DCA is a novel method for evaluating diagnostic tests, prediction models and molecular markers. It combines the mathematical simplicity of accuracy measures, such as sensitivity and specificity, with the clinical applicability of decision analytic approaches. All statistical analyses were performed using SPSS 25.0 software (SPSS Inc., Chicago, IL, U.S.A.) and graph plotting were performed by GraphPad Prism 7.0 (GraphPad Software, U.S.A.). When *P*<0.05, the result was considered to be statistically significant.

### Bioinformatics analysis

In order to evaluate the potential biological processes and pathways of the identified miRNAs, bioinformatics analysis was performed for GO biological process and Kyoto Encyclopedia of Genes and Genomes (KEGG) pathway using the DIANA-miRPath v3.0, a pathway analysis web server for miRNA targets [14].

## Results

### Characteristics of subjects

In the present study, a total of 194 participants (92 EC patients and 102 NCs) were collected to measure differentially expressed circulating miRNAs. The serum samples were separately allocated to four phases: the screening phase, the training phase, the testing phase, and an external validation phase, as depicted in Figure 1. The detailed clinical characteristics of participants are given in Table 1. There was no significant difference in the distribution of age between EC patients and NCs during the four stages (*P*>0.05).

### Expression profiling of miRNAs from the screening phase

In the screening phase, the expression profile of 174 miRNAs was measured by the Exiqon miRCURY-Ready-to-Use PCR-Human-panel-I+II-V1.M in 2 peripheral serum pools from 20 EC patients and 1 pooled sample from 10 female NCs. Candidate miRNAs were selected based on the following criteria: (i) with a *C*_t_ value < 37; (ii) a *C*_t_ value 5 lower than the negative control (No Template Control, NTC); (iii) having at least 1.5-fold altered expression. A total of 29 differentially expressed miRNAs were selected as candidate miRNAs and selected for further evaluation by qRT-PCR (Supplementary Table S2).

### Confirmation of miRNAs by qRT-PCR analysis

**Figure 2:**
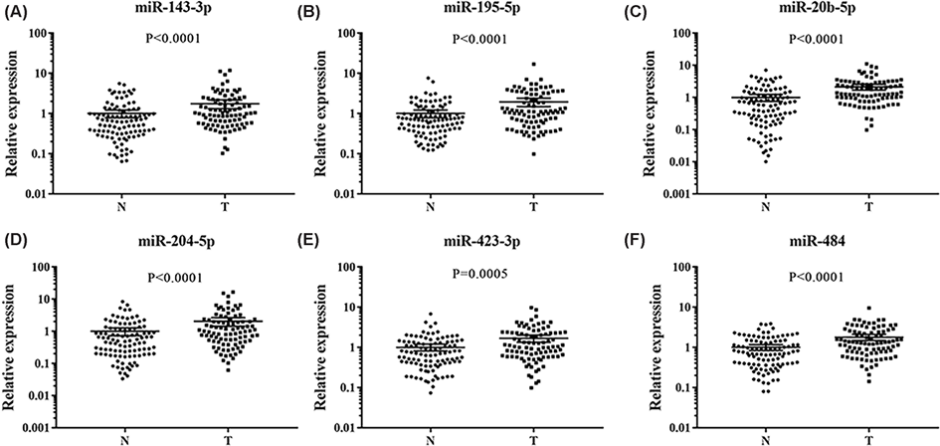
Expression levels of the six miRNAs in the serum of 92 EC patients and 102 female NCs N: normal controls; T: tumor. Horizontal line: mean with 95% CI. (**A**) miR-143-3p: *P*<0.0001, N: 1, 95% CI: 0.78–1.22; T: 1.75, 95% CI: 1.32–2.18. (**B**) miR-195-5p: *P*<0.0001, N: 1, 95% CI: 0.78–1.22; T: 1.95, 95% CI: 1.48–2.42. (**C**) miR-20b-5p: *P*<0.0001, N: 1, 95% CI: 0.76–1.24; T: 2.12, 95% CI: 1.69–2.54. (**D**) miR-204-5p: *P*<0.0001, N: 1, 95% CI: 0.73–1.27; T: 2.03, 95% CI: 1.43–2.64. (**E**) miR-423-3p: *P*=0.0005, N: 1, 95% CI: 0.81–1.19; T: 1.68, 95% CI: 1.33–2.02. (**F**) miR-484: *P*<0.0001, N: 1, 95% CI: 0.84–1.16; T: 1.79, 95% CI: 1.48–2.10. Abbreviation: CI, confidence interval.

In the following training phase, the study tested the 29 candidate miRNAs between 21 EC patients and 24 female NCs. A total of 13 miRNAs with differential expression were defined (miR-92a-3p, miR-145-5p, miR-423-3p, miR-151a-3p, miR-195-5p, miR-204-5p, miR-20b-5p, miR-484, miR-590-5p, miR-92b-3p, miR-125b-5p, miR-143-3p, miR-29c-3p). Then the 13 miRNAs were validated in the testing phase (41 EC patients and 48 female NCs), and 6 miRNAs were obtained ultimately (miR-143-3p, miR-195-5p, miR-20b-5p, miR-204-5p, miR-423-3p, and miR-484). The results of the other miRNAs were excluded from the panel as supplementary material (Supplementary Table S3). The EC patients above were all from the First Affiliated Hospital of Nanjing Medical University. In order to verify the capacity of these six identified miRNAs as the serum characteristics of EC patients, an independent cohort study was conducted with 30 EC patients and 30 NCs from the Women’s Hospital of Nanjing Medical University. The result suggested that six serum miRNAs may be potential diagnostic biomarkers for EC patients (Figure 2).

### Diagnostic value of identified miRNAs in serum

We used ROC curve analysis to evaluate the performance of the three miRNAs in identifying EC patients from NCs. The AUCs for identifying EC patients versus healthy controls were 0.677 (95% confidence interval (CI): 0.602–0.751) for miR-143-3p, 0.669 (95% CI: 0.593–0.745) for miR-195-5p, 0.756 (95% CI: 0.689–0.823) for miR-20b-5p, 0.668 (95% CI: 0.592–0.743) for miR-204-5p, 0.689 (95% CI: 0.611–0.767) for miR-423-3p, and 0.644 (95% CI: 0.566–0.722) for miR-484 in the combined cohorts between 92 EC patients and 102 female NCs, respectively (Supplementary Figure S1).

**Figure 3:**
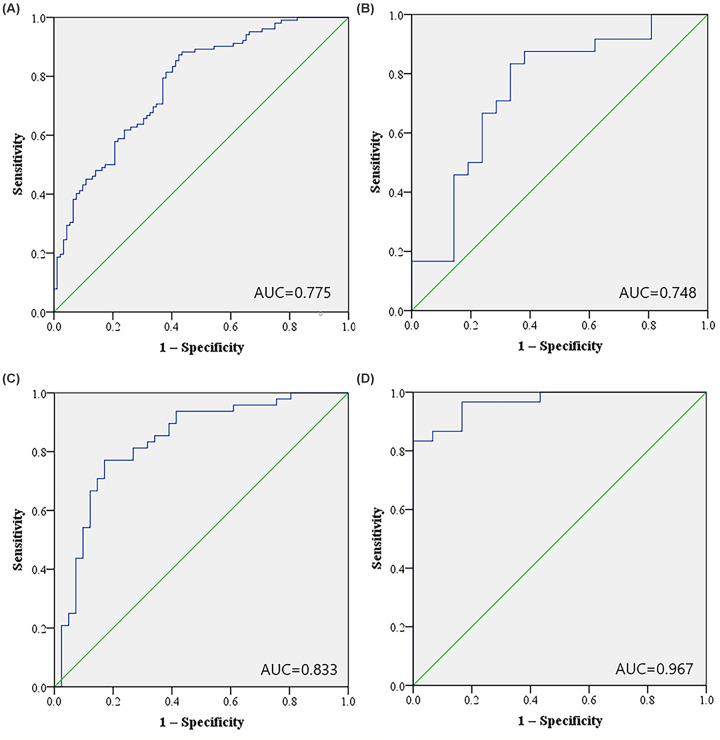
ROC curves for the 6-miRNA panel to discriminate EC patients from NCs (**A**) The combined data of training and testing stages: AUC = 0.775, 95% CI: 0.710–0.840, *P*<0.001, Sensitivity = 78.4%, Specificity = 63.0%. (**B**) The training stage: AUC = 0.748, 95% CI: 0.599–0.897, *P*=0.004, Sensitivity = 83.3%, Specificity = 66.7%. (**C**) The testing stage: AUC = 0.833, 95% CI: 0.745–0.921, *P*<0.001, Sensitivity = 77.1%, Specificity = 82.9%. (**D**) The external validation stage: AUC = 0.967, 95% CI: 0.928–1.000, *P*<0.001, Sensitivity = 83.3%, Specificity = 100%.

**Figure 4:**
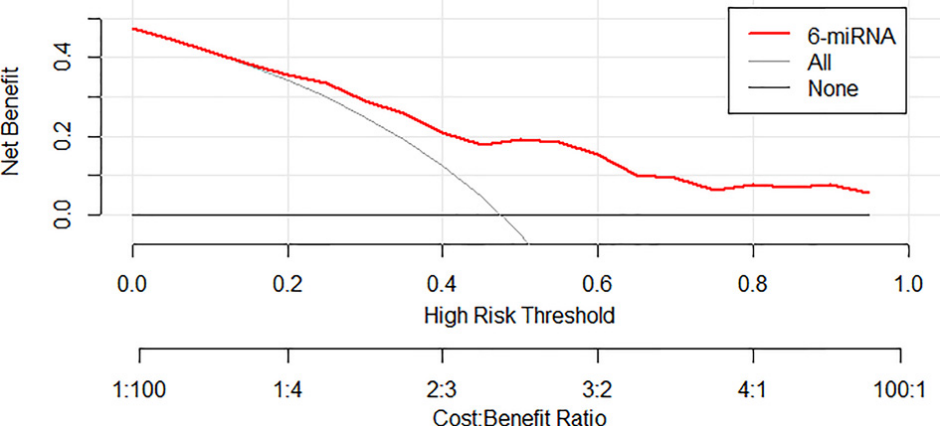
The result of DCA of the 6-miRNA signature

We then combined the six miRNAs together and constructed a 6-miRNA panel in serum to discriminate EC patients from health females. The probability of EC was evaluated using a multiple logistic regression model and calculated using the following formula: Logit (P) = 1.715 − 0.314 × miR-143-3p − 0.309 × miR-195-5p − 0.337 × miR-20b-5p − 0.443 × miR-204-5p + 0.424 × miR-423-3p − 0.443 × miR-484. As shown in Figure 3A, the AUC of the panel was 0.775 (95% CI: 0.710−0.840, *P*<0.000), representing a significant improvement compared with each single miRNA marker. As shown in the DCA curve, when the threshold is greater than 15%, the 6-miRNA signature owe better diagnostic performance (Figure 4). Meanwhile, the diagnostic performance of the 6-miRNA panel was also evaluated in the training, testing and external validation phases and the AUCs were 0.748 (95% CI: 0.599–0.897, *P*=0.004), 0.833 (95% CI: 0.745–0.921, *P*<0.000), and 0.967 (95% CI: 0.928–1.000, *P*<0.000), respectively (Figure 3B–D).

### Relationship between the miRNAs and clinicopathological parameters

In addition, we analyzed the association between the identified miRNAs and the clinicopathological parameters of 92 EC patients. According to FIGO stage classification, 92 EC patients were classified as early (I) and late (II + III + IV) stage. Almost all six identified serum miRNAs were consistently up-regulated in each subgroup compared with those in NCs (Supplementary Figure S2). Compared with the NC group, the ROC curves of early (I) and late (II + III + IV) were 0.769 and 0.844, respectively, which proved that the 6-miRNA marker performs well in distinguishing EC patients with different FIGO stage from NCs (Table 2). We also performed a subgroup analysis based on histological grade. Compared with serum miRNAs in NCs, almost all six identified serum miRNAs in each subgroup were up-regulated (*P*<0.05; Supplementary Figure S3). Subgroup ROC curve analysis further compared EC patients with different levels of differentiation and normal control (NC) groups. The 6-miRNA panel had a reliable performance in distinguishing EC patients at any stage from healthy people, with AUCs of 0.773 (95% CI: 0.679–0.867, sensitivity = 62.9%, specificity = 81.4%), 0.766 (95% CI: 0.685–0.848, sensitivity = 69.8%, specificity = 70.6%), and 0.874 (95% CI: 0.787–0.961, sensitivity = 91.7%, specificity = 71.6%) for patients at histological grades I, II, III, and IV, respectively (Table 2). On the other hand, there is no significant difference between patient subgroups, indicating that the overall marker expression does not change with changes in patient characteristics, and the differential expression is relatively stable (Supplementary Figure S3).

**Table 2:** The AUCs of the 6-miRNA panel for discriminating EC patients at different clinicopathological parameters

Clinicopathological	AUC	95% CI	Sensitivity (%)	Specificity (%)
TNM, Stage I	0.769	0.701–0.837	60.2	84.3
TNM, Stage II–IV	0.844	0.719–0.970	66.7	92.2
Histological grade, G1	0.773	0.679–0.867	62.9	81.4
Histological grade, G2	0.766	0.685–0.848	69.8	70.6
Histological grade, G3	0.874	0.787–0.961	91.7	71.6

### External validation in public databases

**Figure 5:**
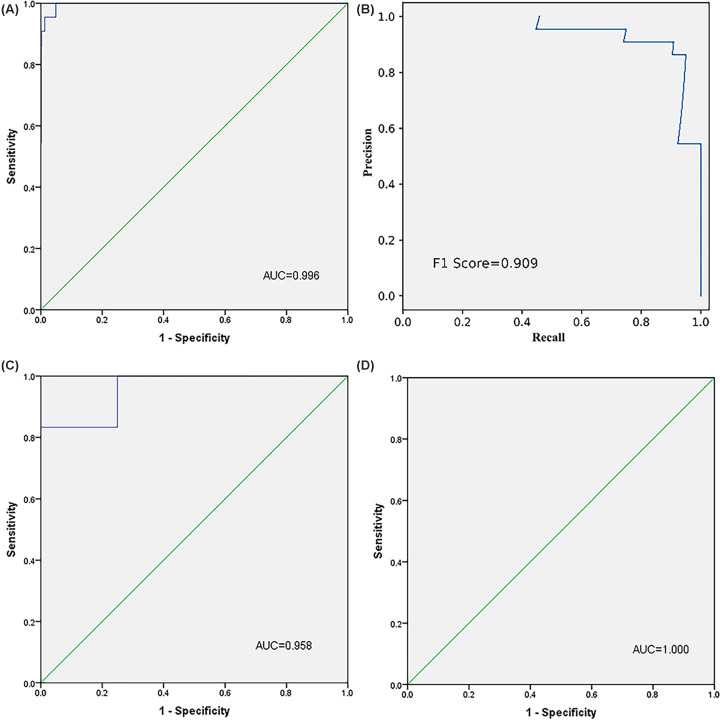
External validation of the 6-miRNA signature in TCGA-UCEC (A,B), GSE35794 (C), and GSE25405 (D) datasets (A) ROC curves of the 6-miRNA signature to distinguish EC tumor tissues from normal tissues in TCGA-UCEC. AUC = 0.996, 95% CI: 0.992–1.000, *P*<0.05, Sensitivity = 100%, Specificity = 95.1%. (B) The Precision-Recall Curves of the 6-miRNA signature to distinguish EC tumor tissues from normal tissues in TCGA-UCEC. The F1 score = 0.909; (C) ROC curves of the 6-miRNA signature to distinguish EC patients from healthy people in GSE35794. AUC = 0.958, 95% CI: 0.868–1.000, *P*<0.05, Sensitivity = 83.3%, Specificity = 100%. (D) ROC curves of the 6-miRNA signature to distinguish EC patients from healthy people in GSE25405. AUC = 1.000, 95% CI: 1.000–1.000, *P*<0.05, Sensitivity = 100%, Specificity = 100%.

The diagnostic value of the 6-miRNA signature was further verified in the three public datasets: The Cancer Genome Atlas Uterine Corpus Endometrial Carcinoma (TCGA-UCEC) dataset (http://cancergenome.nih.gov/), GSE35794 dataset, and GSE25405 dataset. In the TCGA-UCEC dataset, the 6-miRNA marker performed well in distinguishing EC tumor tissues (*n*=531) and normal tissues (*n*=22). The corresponding AUC of the 6-miRNA signature was 0.996 (95% CI: 0.992–1.000, *P*=0.002, sensitivity: 100%, specificity: 95.1%) (Figure 5A). The Precision-Recall Curves analysis on the TCGA data, and the F1 score was 0.909 (Figure 5B). In GSE35794, the AUC of the 6-miRNA panel used to distinguish EC patients from healthy people was 0.958 (95% CI: 0.868–1.000, *P*=0.046, sensitivity: 83.3%, specificity: 100%) (Figure 5C). In GSE25405, the AUC of the 6-miRNA panel used to distinguish EC patients from healthy people was 1.000 (95% CI: 1.000–1.000, *P*<0.001, sensitivity: 100%, specificity: 100%) (Figure 5D). In summary, the diagnostic value of the identified signature was successfully verified in the three public datasets.

### Comparison with previously identified signatures

In order to further verify the diagnostic capabilities of the 6-miRNA signature, we compare it with the six previously reported signatures which can similarly distinguish EC patients from healthy people. As shown in Table 3, compared with other markers, the 6-miRNA marker has the highest AUC value in the TCGA-UCEC and GSE25405, and only slightly worse than Wang et al*.* [16] and Tsukamoto et al*.* [17] in GSE35794 (Supplementary Figure S4). In conclusion, the 6-miRNA panel identified in the present study is superior to other miRNA biomarkers in the diagnosis of EC [10–12,15–17].

**Table 3:** EC diagnostic capability of the signature and previously identified biomarkers (presented as the results of ROC curve analysis using the data from TCGA and GEO datasets)

Study	Year	Biomarker	TCGA			GSE25405			GSE35794		
			AUC	Sensitivity (%)	Specificity (%)	AUC	Sensitivity (%)	Specificity (%)	AUC	Sensitivity (%)	Specificity (%)
**The present study**	**2020**	**6-miRNA panel**	**0.996**	**100.0**	**95.1**	**1.000**	**100.0**	**100.0**	**0.958**	**83.3**	**100.0**
Ritter et al. [10]	2020	miR-484 and miR-23a	0.858	72.9	86.4	0.612	56.4	71.4	0.847	77.8	75.0
Jia et al. [11]	2013	miR-222, miR-223, miR-186, and miR-204	0.865	81.3	81.8	0.864	87.2	85.7	0.806	77.8	75.0
Torres et al. [15]	2013	miR-449a and miR-1228	0.706	63.8	63.6	0.762	66.7	85.7	0.889	77.8	75.0
Montagnana et al. [12]	2017	miR-222, miR-223, miR-186, and miR-204	0.865	81.3	81.8	0.864	87.2	85.7	0.806	77.8	75.0
Wang et al. [16]	2014	miR-15b, miR-27a, and miR-223	0.847	70.9	86.4	0.835	84.6	85.7	1.000	100.0	100.0
Tsukamoto et al. [17]	2014	miR-135b, miR-205, miR-30a-3p, miR-21	0.950	87.2	100.0	0.982	97.4	100.0	1.000	100.0	100.0

### Evaluation of miRNA expression in tissues and serum exosomes

**Figure 6:**
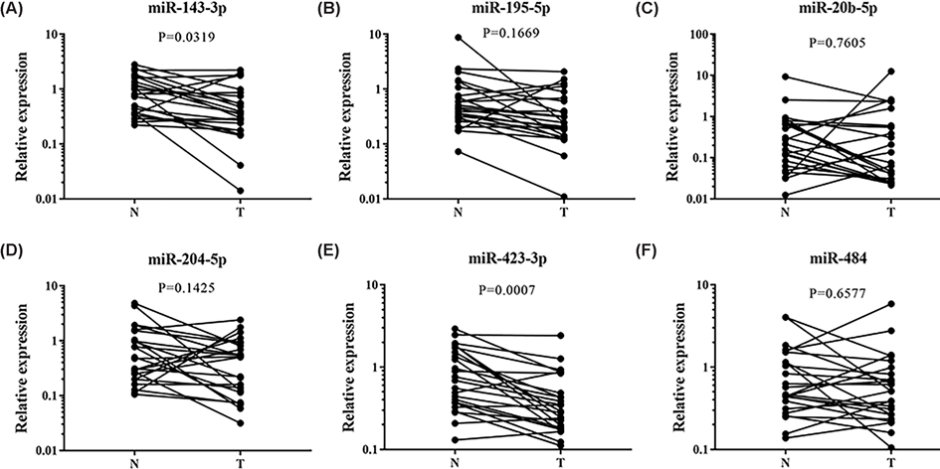
Expression of the six miRNAs in the tumor tissues of 24 pairs of EC patients N: normal controls; T: tumor. (**A**) miR-143-3p: *P*=0.0319. (**B**) miR-195-5p: *P*=0.1669. (**C**) miR-20b-5p: *P*=0.7605. (**D**) miR-204-5p: *P*=0.1425. (**E**) miR-423-3p: *P*=0.0007. (**F**) miR-484: *P*=0.6577.

**Figure 7:**
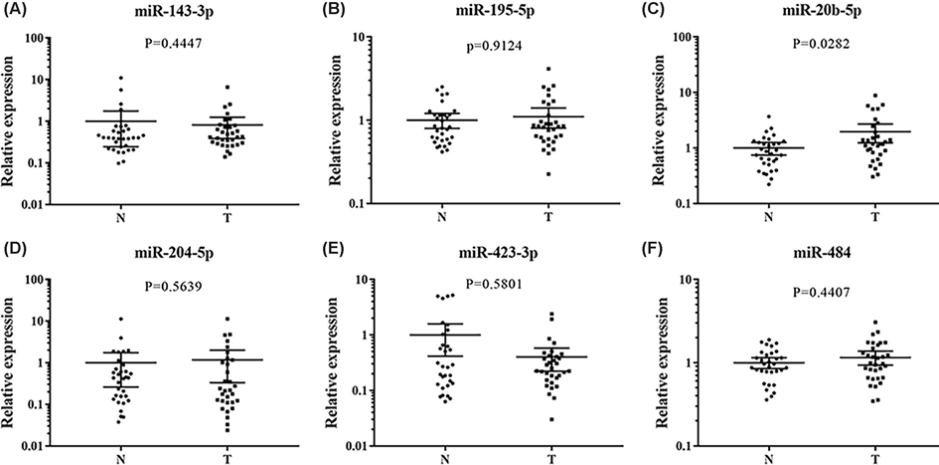
Expression of the six miRNAs in the serum exosomes of 32 EC and 32 NCs N: normal controls; T: tumor. Horizontal line: mean with 95% CI. (**A**) miR-143-3p: *P*=0.4447, N: 1, 95% CI: 0.24–1.76; T: 0.81, 95% CI: 0.38–1.24. (**B**) miR-195-5p: *P*=0.9124, N: 1, 95% CI: 0.80–1.20; T: 1.10, 95% CI: 0.80–1.40. (**C**) miR-20b-5p: *P*=0.0282, N: 1, 95% CI: 0.74–1.26; T: 1.97, 95% CI: 1.24–2.70. (**D**) miR-204-5p: *P*=0.5639, N: 1, 95% CI: 0.26–1.74; T: 1.17, 95% CI: 1.033–2.01. (**E**) miR-423-3p: *P*=0.5801, N: 1, 95% CI: 0.41–1.59; T: 0.40, 95% CI: 0.22–0.58. (**F**) miR-484: *P*=0.4407, N: 1, 95% CI: 0.85–1.15; T: 1.16, 95% CI: 0.93–1.38.

The expression patterns of the 6-miRNA signature were examined in 24 pairs of tissue samples of EC patients and serum exosomes from 32 EC patients and 32 female NCs. MiR-423-3p and miR-143-3p were found significantly down-regulated in tumor tissues compared with their matched controls (Figure 6). In addition, we also evaluated the miRNA expression of 21 pairs of EC tissues and matched normal tissues in the TCGA database. In the TCGA database, the expression of miR-143-3p and miR-195-5p were consistent with our results. However, miR-423-3p and miR-484 were down-regulated in tumor samples (Supplementary Figure S5). As shown in Figure 7, the expression of miR-20b-5p in exosomes was consistent with those in serum, but the other miRNAs showed no significant difference.

### Analysis of identified miRNAs in bioinformatics databases

We use the miRNA pathway analysis web server DIANA-mirPath v3.0 to predict the possible functions of target miRNAs (miR-143-3p, miR-195-5p, miR-20b-5p, miR-204-5p, miR-423-3p, and miR-484). According to KEGG analysis, these identified miRNAs might have some relationship with several tumor-related signaling pathways, including p53 signaling pathway, fatty acid metabolism, and cell cycle (Supplementary Figure S6A). The results of GO classification indicate that the 6-miRNA signature participates in the process of cell protein modification, binds to cytoskeleton proteins, and has nucleic acid binding transcription factor activity (Supplementary Figure S6B). P53 mutation plays a crucial role in the occurrence of EC. p53-R248Q targets the proteasome activator REGγ to promote EC progression. The increased expression of p53 is closely related to the high pathological grade of EC specimens and lymph node metastasis [18]. Cellular proliferation, a common feature of all cancers, requires fatty acids to synthesize membranes and signaling molecules. The reprogramming of fatty acid metabolism can stimulate the proliferation and invasion of cancer cells, by providing phospholipids and cholesterol for the synthesis of cancer cell membranes, and provide energy sources through β oxidation, leading to the malignant progression of cancer [19]. The results indicate the possible role of candidate miRNAs in the pathogenesis of EC. Heatmaps of the targeted pathways are shown in Supplementary Figure S6.

## Discussion

EC is one of the most common malignant tumors in females worldwide. From a clinical perspective, specific miRNAs can serve as biomarkers for EC diagnosis and therapy. Tumor-derived miRNAs in blood have been established as effective markers for cancer diagnosis and prognosis prediction [16]. However, due to multiple miRNA quantitative platforms, study subjects, or analytical methods, these studies often produce conflicting results.

Briefly, we carefully established a four stepwise procedure to identify a serum miRNA signature for EC. Exiqon miRNA qPCR panel was utilized to conduct miRNA profile in the initial screening phase. Three phases were performed to control the false positive rate (training phase, testing phase, and external validation phase) using qRT-PCR. Compared with a single miRNA, the complex patterns of miRNAs could provide more reliable information about disease conditions. Based on the abnormally expressed miRNAs screened out, a panel was constructed using logistic regression, and we daringly hypothesized that the miRNA panel signature might become a potential biomarker for EC detection.

In the present study, we found a specific circulating miRNA panel in EC patients, which consisted of miR-143-3p, miR-195-5p, miR-20b-5p, miR-204-5p, miR-423-3p and miR-484. The 6-miRNA marker can distinguish cancer from normal, different subgroups from normal. It has been verified in other databases and is superior to some previously discovered markers.

Data showed that miR-143-3p might act as a novel tumor-suppressive factor by regulating tumorigenesis and progression. The worse prognosis in EC was also reported to be associated with miR-143-3p [20]. A previous study showed that miR-143-3p could inhibit cell proliferation and metastasis of EC by mitogen-activated protein kinase (MAPK1) [21]. Another study identified that progesterone 4 (P4) could regulate miR-143-3p expression in endometrial epithelial cells [22]. Growing evidence also demonstrated that miR-423-3p could play essential roles in tumorigenesis and progression. It was identified that miR-423-3p might inhibit cisplatin-induced apoptosis from decreasing the sensitivity of EC cell through mediating the expression of Bcl-2 and caspase 3/7 [23]. MiR-195-5p bound signaling pathway target genes involved in oncogenic mechanisms, such as apoptosis, cell proliferation, and was aberrantly expressed in a number of cancers as well [24,25]. Tsukamoto et al. and Jayaraman et al. were in agreement with the result that miR-195-5p showed down-expression in EC tissues [17,26]. MiR-20b-5p as part of the miR-106a/363 cluster, could modulate vascular endothelial growth factor A (VEGFA) transcription by targeting hypoxia-inducible factor 1 A (HIF1A), phosphatase and tensin homolog (PTEN), and activator of transcription 3 (STAT3) [5,27,28]. In Eismann et al.’s study, they observed reduced miR-20b-5p expression levels in EC cells under hypoxic conditions [29]. MiR-20b-5p might act as a tumor inhibitor by impeding MMP-2 expression leading to cell cycle arrest as well as regulative function on oxygen balance [29]. As same with our result, overexpression of miR-204-5p in the serum of EC patients reached statistical significance in Jia et al.’s study [11]. It was believed that miR-204-5p could suppress cancer procedure by promoting apoptosis, conferring resistance to chemotherapy, suppressing epithelial-to-mesenchymal transition (EMT), and self-renewing cancer stem cells. Expression of miR-204-5p was repressed by targeting XRN1 and TRKB in prostate cancer and EC, respectively.

The serum miRNAs for EC detection were also explored in matched tissues to further strengthen the hypothesis we investigated. However, only miR-423-3p in tissues showed a meaningful downward trend. Subsequently, we analyzed the miRNA profiles of 24 pairs of matched tissues of EC patients based on the TCGA dataset. We found miR-423-3p and miR-484 expressed lower in tissues than in adjacent normal tissues from TCGA, while the expression levels of miR-143-3p and miR-195-5p were up-regulated in EC tissues. A reasonable speculation is that there exists a discrepancy of the miRNA expression profiles between Caucasians and Asians. In addition, differences in gene mutation rates between different ethnic groups have been verified in previous research [30].

At the same time, we would like to determine the KEGG pathway of the six miRNAs. Through bioinformatics analysis, we discovered that the overlapping target genes or pathways of each candidate miRNAs were proved to be tightly related to the biological processes of cancer, such as p53 signaling pathway, participation on cancer proteoglycans, and cell cycle. Interestingly, fatty acid metabolism was found to be the target pathway of miR-143-3p, miR-195-5p and miR-484. Fatty acid synthase is a key lipogenic enzyme, which is highly expressed in EC. Given that patients with EC have high rates of obesity, the target should be further investigated as a novel strategy for EC treatment [31]. The GO annotation also indicated that these miRNAs were involved in the biological process of cancer through the cellular protein modification process, cytoskeletal protein binding, nucleic acid binding transcription factor activity, and so on.

Furthermore, the mechanism behind the stability of circulating miRNAs is still elusive. Therefore, in order to better explore the potential forms of miRNAs in serum, the expression of six miRNAs in exosomes was examined. Exosomes are membrane-enclosed extracellular vesicles (40–100 nm), secreted from viable cells into the blood circulation. These vesicles carry plenty of biological molecules, such as proteins, RNAs, DNAs, as well as lipids, and protect these signal molecules away from enzymes [32]. Exosomes are an important mediator of cell-to-cell communication and play a key role in tumor development and metastasis [33]. Recently, exosomal miRNAs in body fluids have been used as one of the circulating biomarkers to detect several cancers [34,35]. In present study, only miR-20b-5p showed a higher expression level in serum-derived exosomes of EC patients, while the others showed no difference. It has been demonstrated that the majority of miRNAs in body fluids may be concentrated in exosomes [36]. On the contrary, Arroyo et al. [37] found that most circulating miRNAs in plasma are cofractionated with Ago2. The hypothesis we proposed was that the majority of extracellular miRNA complexes might be bound to some proteins in serum, such as the Ago2 ribonucleoprotein complex and high-density lipoprotein (HDL) rather than exosome vesicles [37]. The existing forms of miRNAs in the circulation were really complicated and the phenomena were warranted to be explored and validated in the future.

In summary, we identified a 6-miRNA signature in serum (miR-143-3p, miR-195-5p, miR-20b-5p, miR-204-5p, miR-423-3p, and miR-484) which may serve as a promising signature for the accurate diagnosis of EC after the four-phase screening. This result will trigger interest in further intensive research into the elucidation of their functional effects. Although the observations are promising, the results should be confirmed and verified in an independent large-scale cohort before being translated into the clinical setting.

## Conclusion

In conclusion, this experiment has identified and validated a 6-miRNA signature (miR-143-3p, miR-195-5p, miR-20b-5p, miR-204-5p, miR-423-3p, and miR-484) in serum as a potential non-invasive biomarker for EC diagnosis.

## Supplementary Material

Supplementary Figures S1-S6 and Tables S1-S3

## Data Availability

The data that support the findings of the present study are available from the corresponding authors upon reasonable request.

## Abbreviations

AUC: area under the curve
CI: confidence interval
*C*_t_: threshold cycle
DCA: decision curve analysis
EC: endometrial cancer
KEGG: Kyoto Encyclopedia of Genes and Genomes
GO: gene ontology
miRNA: microRNA
NC: normal control
PCR: polymerase chain reaction
qRT-PCR: quantitative real-time polymerase chain reaction
ROC: receiver operating characteristic
TCGA-UCEC: The Cancer Genome Atlas Uterine Corpus Endometrial Carcinoma
