# Supplementary material for: MicroRNA expression profile in serum reveals novel diagnostic biomarkers for endometrial cancer
Source: Biosci Rep. 2021 Jun 16;41(6):BSR20210111. doi: 10.1042/BSR20210111 (PMC8209168; doi:10.1042/BSR20210111)
Supplement: Supplementary Figures S1-S6 and Tables S1-S3 [file BSR-2021-0111_supp.pdf]

Supplementary

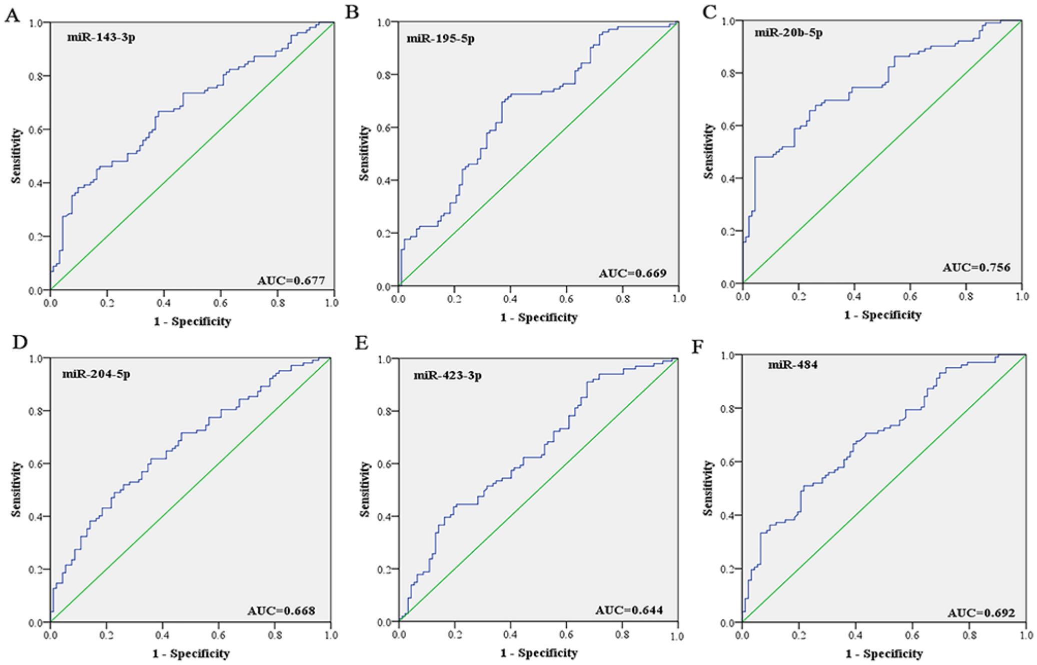

Figure S1: ROC curve analyses of each miRNA to discriminate EC patients from NCs in the combined three phases. A: miR-143-3p; B: miR-195-5p; C: miR-20b-5p; D: miR-204-5p; E: miR-423-3p; F: miR-484.

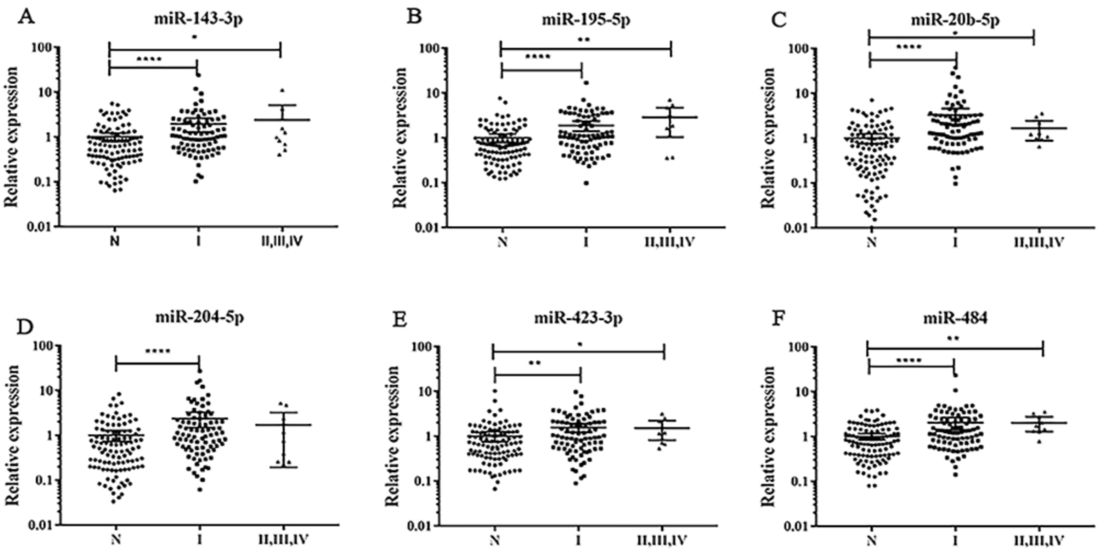

Figure S2: Association of the identified miRNAs with different clinicopathological parameters (FIGO stage). A: miR-143-3p; B: miR-195-5p; C: miR-20b-5p; D: miR-204-5p; E: miR-423-3p; F: miR-484. N: normal controls; *: P < 0.05.

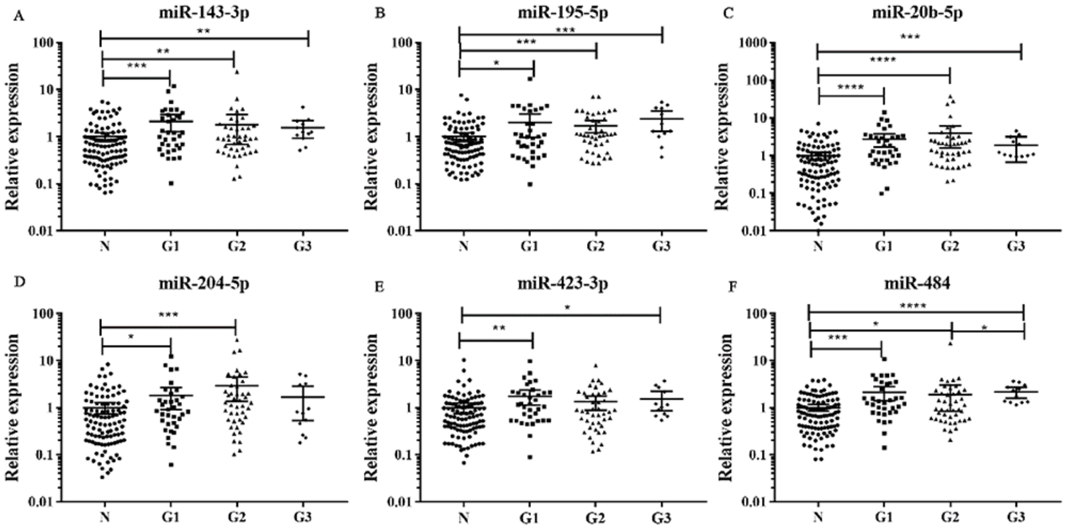

Figure S3: Association of the identified miRNAs with different clinicopathological parameters (histological grade). A: miR-143-3p; B: miR-195-5p; C: miR-20b-5p; D: miR-204-5p; E: miR-423-3p; F: miR-484. N: normal controls; *: $P < 0.05$.

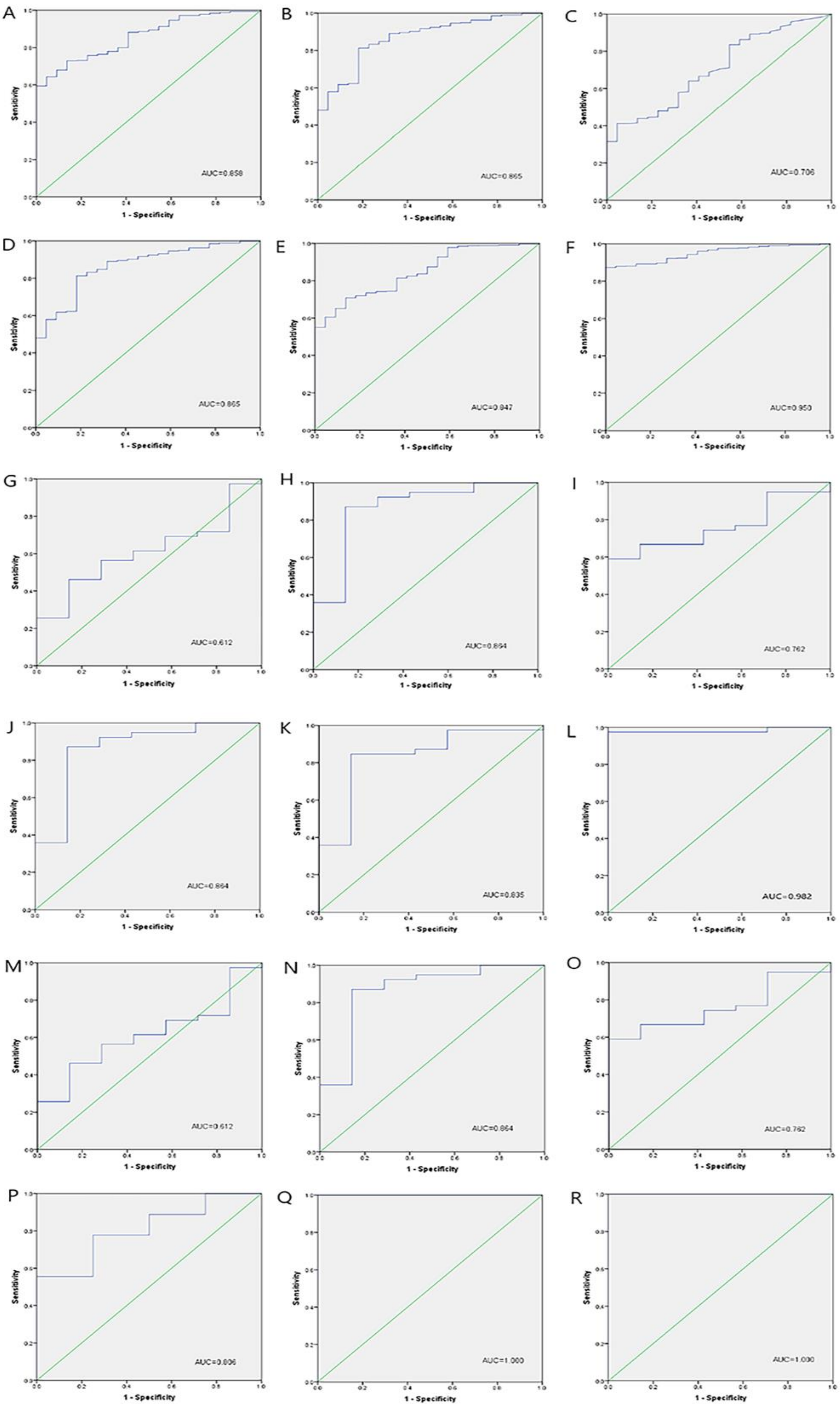

A
AUC=0.858
B
AUC=0.865
C
AUC=0.706
D
AUC=0.865
E
AUC=0.847
F
AUC=0.950
G
AUC=0.612
H
AUC=0.864
I
AUC=0.762
J
AUC=0.864
K
AUC=0.835
L
AUC=0.982
M
AUC=0.612
N
AUC=0.864
O
AUC=0.762
P
AUC=0.806
Q
AUC=1.000
R
AUC=1.000
Sensitivity
1 - Specificity

Figure S4: Endometrial cancer diagnostic capability of the signature and previously identified biomarkers (presented as the results of ROC curve analysis using the data from TCGA and GEO datasets).

TCGA:

(A) Andrea Ritter *et al.*: AUC = 0.858, 95% CI:0.801-0.914, P<0.05, Sensitivity = 72.9 %, Specificity = 86.4%.

(B) Wenhui Jia *et al.*: AUC = 0.865, 95% CI:0.801-0.930, P<0.05, Sensitivity = 81.3%, Specificity = 81.8%.

(C) Anna Torres *et al.*: AUC = 0.706, 95% CI:0.610-0.801, P<0.05, Sensitivity = 63.8%, Specificity = 63.6%.

(D) Martina Montagnana *et al.*: AUC = 0.865, 95% CI:0.801-0.930, P<0.05, Sensitivity = 81.3%, Specificity = 81.8%.

(E) Lin Wang *et al.*: AUC = 0.847, 95% CI:0.785-0.910, P<0.05, Sensitivity = 70.9%, Specificity = 86.4%.

(F) Ozora Tsukamoto *et al.*: AUC = 0.950, 95% CI:0.927-0.972, P<0.05, Sensitivity = 87.2%, Specificity = 100%.

GSE25405:

(G) Andrea Ritter *et al.*: AUC = 0.612, 95% CI:0.420-0.803, P<0.05, Sensitivity = 56.4 %, Specificity = 71.4%.

(H) Wenhui Jia *et al.*: AUC = 0.864, 95% CI:0.698-1.000, P<0.05, Sensitivity = 87.2%, Specificity = 85.7%.

(I) Anna Torres *et al.*: AUC = 0.762, 95% CI:0.617-0.906, P<0.05, Sensitivity = 66.7%, Specificity = 85.7%.

(J) Martina Montagnana *et al.*: AUC = 0.864, 95% CI:0.698-0.1.000, P<0.05, Sensitivity = 87.2%, Specificity = 85.7%.

(K) Lin Wang *et al.*: AUC = 0.835, 95% CI:0.0.669-1.000, P<0.05, Sensitivity = 84.6%, Specificity = 85.7%.

(L) Ozora Tsukamoto *et al.*: AUC = 0.982, 95% CI:0.944-1.000, $P<0.05$, Sensitivity = 97.4%, Specificity = 100%.

GSE35794:

(M) Andrea Ritter *et al.*: AUC = 0.847, 95% CI:0.652-1.000, $P<0.05$, Sensitivity = 77.8 %, Specificity = 75%.

(N) Wenhui Jia *et al.*: AUC = 0.806, 95% CI:0.597-1.000, $P<0.05$, Sensitivity = 77.8%, Specificity = 75%.

(O) Anna Torres *et al.*: AUC = 0.889, 95% CI:0.736-1.000, $P<0.05$, Sensitivity = 77.8%, Specificity = 75%.

(P) Martina Montagnana *et al.*: AUC = 0.806, 95% CI:0.597-1.000, $P<0.05$, Sensitivity = 77.8%, Specificity = 75%.

(Q) Lin Wang *et al.*: AUC = 1.000, 95% CI:1.000-1.000, $P<0.05$, Sensitivity = 100%, Specificity = 100%.

(R) Ozora Tsukamoto *et al.*: AUC = 1.000, 95% CI:1.000-1.000, $P<0.05$, Sensitivity = 100%, Specificity = 100%.

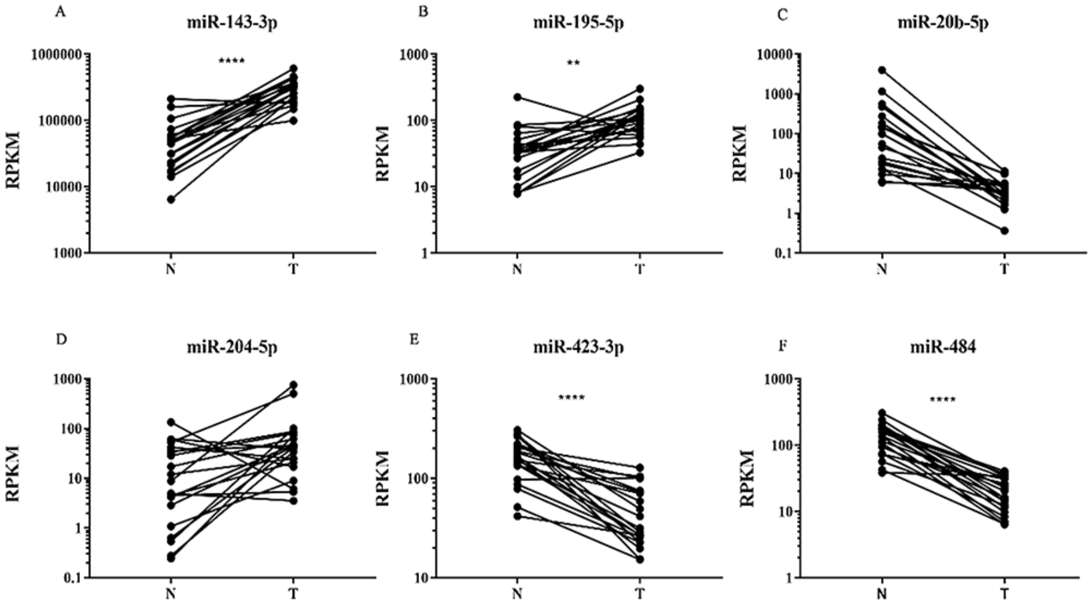

Figure S5: Expression levels of the six miRNAs the tumor tissues of 21 pairs of EC patients in TCGA data. A: miR-143-3p; B: miR-195-5p; C: miR-20b-5p; D: miR-204-5p; E: miR-423-3p; F: miR-484. N: normal controls; *: P < 0.05.

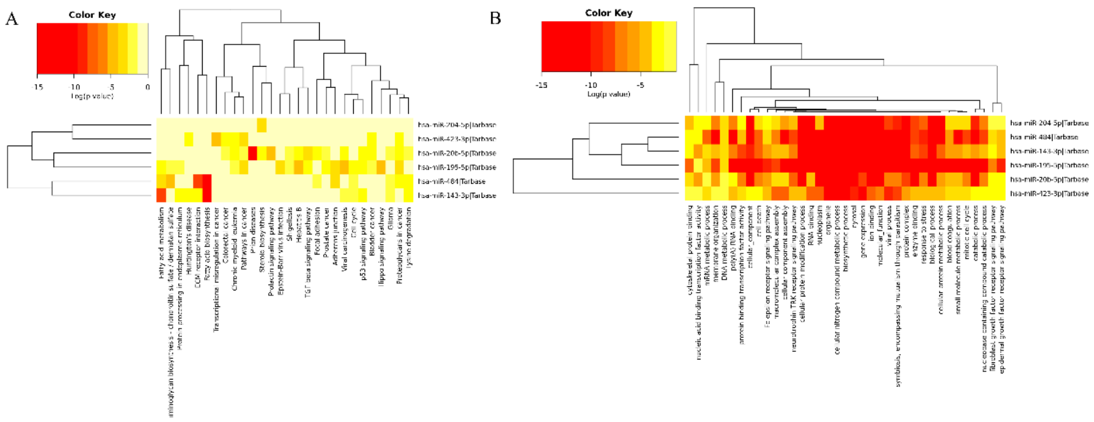

Figure S6 Heat-maps of pathway investigation using KEGG and GO analyses. A: KEGG; B: GO. KEGG: Kyoto Encyclopedia of Genes and Genomes; GO: Gene Ontology.

Table S1: Inclusion and exclusion criteria of cases and controls.

	Cases	Controls
**Inclusion criteria**	(1) Women	(1) Women
	(2) Age 18-75	(2) Age 18-75
	(3) Pathologically diagnosed as endometrial cancer	(3) Clinical confirmation of endometrial cancer-free
	(4) No treatment	(4) Written informed consent
	(5) Written informed consent	
**Exclusion criteria**	(1) History of previous or concomitant malignancies	(1) History of previous or concomitant malignancies
	(2) Other severe systematic diseases that considered to be unsuitable for the study	(2) Other severe systematic diseases that considered to be unsuitable for the study
	(3) History of antitumor therapy	(3) Pregnant or lactating women
	(4) Pregnant or lactating women	(4) Without informed consent
	(5) Without informed consent	

Table S2: Differently expressed miRNAs in the screening phase

miRNA	FC (Exiqon panels)		
	pool1	pool2	Mean fold
hsa-miR-484	1.90	2.02	1.96
hsa-miR-605	4.92	8.68	6.80
hsa-miR-106b-3p	2.15	2.53	2.34
hsa-miR-10a-5p	3.47	3.32	3.39
hsa-miR-10b-5p	6.41	2.40	4.40
hsa-miR-125b-5p	-2.97	-7.60	-5.29
hsa-miR-142-3p	1.78	1.94	1.86
hsa-miR-143-3p	-9.96	-10.26	-10.11
hsa-miR-144-5p	3.69	2.50	3.09
hsa-miR-145-5p	3.68	2.79	3.24
hsa-miR-151a-3p	3.97	2.55	3.26
hsa-miR-190a-5p	5.74	2.65	4.20
hsa-miR-195-5p	6.05	2.35	4.20
hsa-miR-200a-3p	2.24	4.87	3.56
hsa-miR-204-5p	6.87	5.55	6.21
hsa-miR-208a-5p	6.05	8.68	7.36
hsa-miR-20b-5p	5.03	4.84	4.93
hsa-miR-222-3p	2.17	1.56	1.87
hsa-miR-29c-3p	-4.20	-9.00	-6.60
hsa-miR-320b	3.11	2.20	2.66
hsa-miR-335-5p	1.72	1.99	1.85
hsa-miR-361-3p	6.05	8.68	7.36
hsa-miR-409-3p	2.31	1.90	2.10
hsa-miR-423-3p	1.53	1.64	1.59
hsa-miR-500a-5p	2.24	1.80	2.02
hsa-miR-590-5p	1.97	1.58	1.78
hsa-miR-92a-3p	2.17	1.73	1.95
hsa-miR-92b-3p	7.92	4.88	6.40
hsa-let-7c	-3.35	-8.48	-5.91
Abbreviation: FC, fold change.			

Table S3: Expression levels of the identified miRNAs from screening phase but not passed through the training stage and the testing stage (presented as mean±SD; ΔCT).

**miRNA**	**Training stage**				**Testing stage**			
	Cases	Controls	FC	P value	Cases	Controls	FC	P value
let-7c	7.5±1.02	7.5±0.98	1.00	0.653				
miR-106b-3p	10.66±1.47	10.01±1.51	0.64	0.212				
miR-10a-5p	6.44±1.23	5.9±1.68	0.69	0.385				
miR-10b-5p	9.88±1.46	7.9±0.79	0.25	0.597				
miR-125b-5p	7.35±0.96	7.54±1.03	1.14	0.019	5.93±3.45	6.31±2.36	1.3	0.769
miR-142-3p	8.3±1.34	7.74±1.04	0.68	0.175				
miR-144-5p	13.87±1.44	14.32±0.78	1.36	0.247				
miR-145-5p	6.27±2.48	7.11±2.46	1.79	0.003	6.21±1.93	6.41±1.11	1.2	0.067
miR-151a-3p	7.99±1.6	8.49±1.22	1.42	0.011	7.46±2.64	8.22±1.45	1.7	0.059
miR-190a-5p	13.06±1.08	13.63±1.29	1.48	0.561				
miR-200a-5p	11.52±0.99	11.54±1.24	1.01	0.673				
miR-208a-5p	12.62±3.41	12.36±2.55	0.84	0.379				
miR-222-3p	6.01±1.03	6.16±0.68	1.11	0.286				
miR-29c-3p	9.63±1.18	10.11±1.26	1.39	0.021	10.81±1.95	11.52±1.21	1.6	0.213
miR-320b	1.29±0.99	0.9±1.2	0.76	0.174				
miR-335-5p	13.55±1.57	13.05±1.2	0.71	0.716				
miR-361-3p	11.25±3.56	11.25±3.64	1.00	0.801				
miR-409-3p	9.5±0.92	9.08±0.76	0.75	0.673				
miR-500a-5p	11.05±1.21	10.57±1.41	0.72	0.598				
miR-590-5p	11.41±1.4	11.96±1.24	1.47	0.013	10.83±2.95	12.02±1.82	2.3	0.052
miR-605	11±1.61	10.95±1.54	0.97	0.587				
miR-92a-3p	2.62±1.1	3.12±0.9	1.41	0.019	1.89±2.38	3.2±1.14	2.5	0.061
miR-92b-3p	8.25±1.2	8.71±0.78	1.38	0.006	7.9±2.18	8.44±1.3	1.5	0.078
